# Using Human‐Centred Design to Codesign Patient Engagement Tools With a Patient Advisory Council: Successes and Challenges

**DOI:** 10.1111/hex.70230

**Published:** 2025-03-15

**Authors:** Madelyn Knaub, Santiago Leon, Surakshya Pokharel, Veronika Kiryanova, Kim Giroux, Prachi Khanna, Anna Rychtera, D'Arcy Duquette, Pamela Mathura, Nancy Verdin, Anshula Ambasta

**Affiliations:** ^1^ Ward of the 21st Century University of Calgary Calgary Canada; ^2^ Department of Medicine, Cumming School of Medicine University of Calgary Calgary Canada; ^3^ Alberta Strategy for Patient Oriented Research SUPPORT Unit (AbSPORU), O'Brien Institute—Alliances University of Calgary Calgary Canada; ^4^ London School of Hygiene and Tropical Medicine London UK; ^5^ Michael Smith Health Research BC Vancouver Canada; ^6^ Western University London Canada; ^7^ Department of Medicine University of Alberta Edmonton Canada; ^8^ Department of Anesthesia, Pharmacology and Therapeutics, Therapeutics Initiative University of British Columbia Vancouver Canada

**Keywords:** codesign, human centred design, patient advisory council, patient engagement

## Abstract

**Background:**

Co‐build is one of the four pillars of the Patient Engagement Framework from the Canadian Institutes of Health Research Strategy for Patient Oriented Research. Collaborating with Patient Research Partners (PRPs) using co‐build approaches can enhance the applicability of healthcare tools produced. Human Centred Design (HCD), a problem‐solving methodology focused on creating functional solutions for users, offers a promising approach to co‐building patient engagement tools.

**Objective:**

To describe the process of using a HCD approach to co‐build patient engagement tools with PRPs and to identify successes and challenges encountered.

**Methods:**

A HCD working group was formed from a Patient Advisory Council (PAC) that supports a research program to optimize laboratory test ordering in hospitalized patients. The HCD working group included nine PRPs, two patient engagement team members, and a HCD specialist. The working group employed the Double Diamond 4D design methodology: Discover, Define, Design, and Deliver, along with patient engagement principles of mutual respect, inclusiveness, support, and co‐build. At the conclusion of the HCD process, we conducted a semi‐structured debrief session to obtain perspectives on challenges and successes from all working group members. These were then summarized and collated iteratively with feedback from the group members.

**Results:**

The working group met 31 times in 12 months and co‐developed three patient engagement tools (an infographic, a video, and a website) to educate and engage hospitalized patients about the bloodwork process. HCD working group members valued the diverse and inclusive environment within the group, the available enrichment opportunities in HCD and qualitative research, and presence of patient engagement team members. Challenges noted included delays in timelines due to difficulties with consensus‐building and redundancy in discussion topics.

**Conclusion:**

HCD approaches can be effectively combined with the principles of patient engagement to facilitate co‐building with PRPs in healthcare. Future research is required to further the evidence for these strategies and their application in co‐building processes, including use of clear project mapping and timelines and transparent consensus‐building approaches.

**Patient or Public Contribution:**

A PAC that consisted of nine PRPs guided this study. PRPs collaborated throughout the study. The current six PRPs were involved in the decision to write and are co‐authors on this manuscript. PAC members had participated equally in the conduct of a prior qualitative study to understand patient needs about bloodwork processes in hospitals. With the guidance of a HCD specialist, PRPs contributed to decisions on content, wording, and imagery for the tools. The PAC members are currently collaborating on a study to implement these tools in hospitals and to evaluate the utility from a patient perspective.

## 1 Introduction

A growing body of evidence suggests that when patients are involved in research, patient needs are better met and the outcomes that matter to patients are better captured [1, 2, 3]. The Canadian Institutes of Health Research (CIHR) has outlined four major principles for patient engagement through the Strategy for Patient Oriented Research (SPOR) Patient Engagement Framework [4]. These guiding principles are inclusiveness, support, mutual respect, and co‐build. Co‐build is defined as a collaborative process where patients, researchers and healthcare professionals work together to identify problems and gaps, prioritize research, and create and implement solutions [4]. In the literature, co‐build has been accomplished using participatory research, focus groups, interviews, surveys and workshops [5, 6].

Human Centred Design (HCD) is an empathetic approach [7, 8] to create functional and seamless solutions that resonate with the users' needs, preferences, behaviours, and perceptions [7]. The process involves taking the users through every step of the design and consistently iterating back with the users to ensure that their needs are being met [8]. HCD can be applied to both digital and physical products, including service experiences [7]. It has emerged as an innovative method for developing impactful solutions fostering greater user satisfaction and enhanced usability [9]. Considering that the goal of co‐build in health research is to serve patients by collaborating with them, and the HCD approach aims to involve end‐users in product development, these processes are complementary.

HCD is increasingly recognized as a valuable method to address challenges in the healthcare system [7, 10, 11], especially in meeting a growing demand for person‐centred care that is individualized, optimized, and strives for continuous innovation [8, 10]. HCD has the potential to fill these gaps [8, 10, 12], and several studies have reported its successful application in healthcare settings [2, 8, 13, 14]. However, operationalizing HCD methodologies within health research can be challenging, as HCD originated outside the healthcare context, and failure of application within healthcare implementation could lead to serious negative outcomes for patients [8, 15]. While there are examples of HCD being applied in healthcare settings to optimize end‐user experiences (i.e., experiences of patients) [10], there is limited research on whether patients are actively invited to collaborate in the design process. Furthermore, there is a notable gap in the current literature, as HCD has not been described in the context of co‐building with patient research partners (PRPs), where HCD principles are combined with those of patient engagement.

‘Re‐Purposing the Ordering of “Routine” laboratory Tests’ (RePORT) is a patient‐oriented project aimed at reducing the repetitive use of routine laboratory tests in hospitalized patients through the development and implementation of healthcare provider and patient engagement tools. The RePORT team is guided by a Patient Advisory Council (PAC) comprised of PRPs, academic researchers, specialists, and a patient engagement team. We have previously published on how we applied the principles of SPOR's Patient Engagement Framework [4] to establish and sustain the PAC [16] and evaluated patient engagement within the PAC [17]. Additionally, the PAC has previously co‐led a qualitative study to understand patient and family/caregiver experiences and perspectives on blood testing in hospitals [18]. This manuscript herein describes how we applied the findings from the qualitative study to authentically co‐build patient engagement tools with PRPs using a HCD approach combined with patient engagement principles, and we share our lessons learned from this process.

## 2 Methods

### 2.1 HCD Working Group Composition

At the time of the study, the PAC was comprised of nine patient research partners (PRPs), three members from the Alberta SPOR Support Unit [19] (AbSPORU) Patient Engagement Team, and seven researchers. The research team included one internal medicine physician (the principal investigator), one quality improvement scientist, one HCD specialist, two resident physicians, and two research associates. Some PAC members volunteered to join a smaller working group, called the HCD working group, which consisted of nine PRPs, two AbSPORU [19] patient engagement team members, one research associate, and one HCD specialist. All HCD working group members were active members of the PAC throughout the process. The PAC was guided by the Terms of Reference (TOR), a co‐developed document by all PAC members, created at the PAC formation and revised yearly by the PAC (Supporting Information S1: Appendix Item 1). The TOR outlines objectives, expectations, functionality, how to step down from the PAC, and much more [16]. The HCD working group followed the guidelines set by the TOR. Members determined their self‐capacity for engagement and if a member needed to step down or step back, they could do so at any time.

### 2.2 HCD Working Group Process

The working group used the Double Diamond model that incorporates four stages known as the 4Ds: (1) Discover, (2) Define, (3) Develop, and (4) Deliver [20]. This established strategy provided clear direction and structure for the co‐building process [20]. In each stage of the Double Diamond model, we intentionally incorporated the CIHR principles of patient engagement [4].

**Figure 1:**
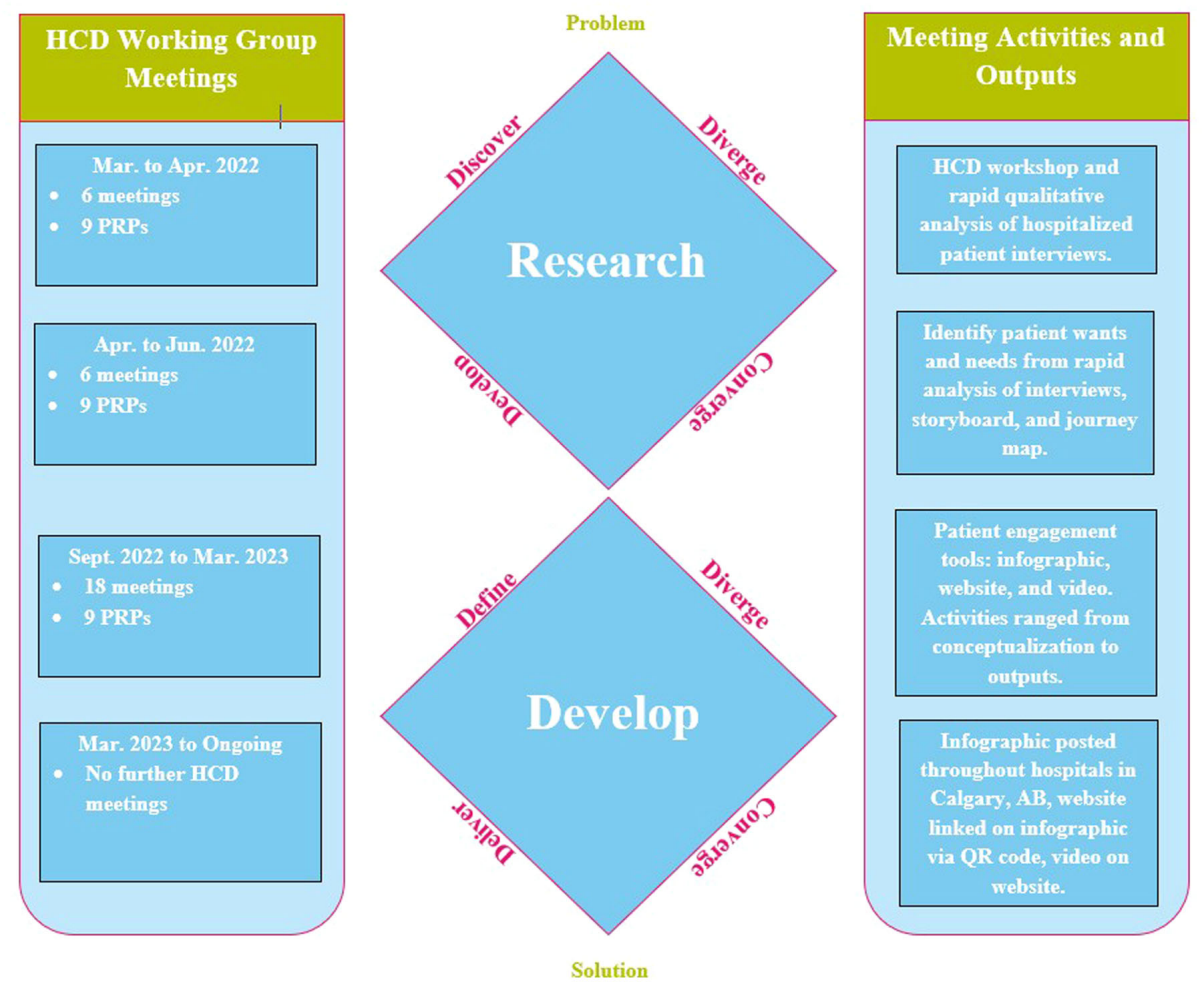
Human centred design working group timeline and double diamond model.

The first stage, *discover*, is when initial research is empathetically gathered about user needs [20]. The *discover* stage began with interviews with patients hospitalized within the past 12 months to obtain a holistic picture of their needs and experiences during the bloodwork process [18] (Figure 1). Semi‐structured interviews were used to allow for empathetic flexibility and adaptation. The interview guide was co‐developed with PRPs. All interviews were conducted over the phone or via Zoom [21] and were audio‐recorded. Twelve out of sixteen interviews were co‐facilitated by PRPs, alongside an academic researcher. PRPs had received training in conducting interviews and analysing qualitative data beforehand [16, 18].

Stage two, *define*, is where data from discovery is analysed to frame the design challenge [20]. The *define* stage was operationalized in this study as qualitative analysis of interview data (Figure 1). While detailed thematic analysis was conducted and reported separately [18], rapid analysis was used to support the codesign of patient engagement tools by PRPs. Rapid analysis is a qualitative research method that is less time intensive than thematic analysis as it involves extracting key insights directly from audio recordings and linking them to quotes, while maintaining rigor in meeting evaluation objectives [22, 23, 24]. This technique is taken from the Consolidated Framework for Implementation Research [22], which focuses on spoken interview data, eliminating the need for transcript evaluation and enabled PRPs to directly work with qualitative data [22, 23].

The third stage in the Double Diamond model, *develop*, is where solutions are developed from the analysed data [20]. For the *develop* stage, the working group decided to develop a set of patient engagement tools addressing needs identified earlier (Figure 1). Weekly recurring sessions with the PRPs and the HCD specialist were held to iteratively develop and refine the patient engagement tools. Some of the updates involved design elements like content, colour theory, themes, iconography, information architecture, interaction flows, usability, and accessibility.

Finally, the fourth stage, *deliver*, is where the solution is delivered to the target population [20]. The developed solution is refined in this stage through further iterations. The *deliver* stage began with sharing one of the patient engagement tools with the participants from the qualitative study. The tools were tested and feedback was collected to refine them before broader dissemination to hospitalized patients. The local health authority was also provided with the infographic before dissemination, and further revisions were made based on feedback and requirements (Figure 1). A formal evaluation of these tools is underway.

After completion of the HCD working group activities, members of the HCD working group met with the principal investigator A.A., and the research associate M.K., to debrief and share their experiences. This debrief session was held to gain insights on the experiences of HCD working group members. This session had guiding questions developed by M.K. and A.A. (Supporting Information S1: Appendix Item 2). The discussion was facilitated by M.K. and A.A. M.K. took detailed notes on the responses of working group members. The session was not recorded to facilitate open and honest feedback. For 1 week after the session, working group members had the opportunity to further respond to the questions posed in the debrief session in a Google Doc [25], if they felt they wanted to share more about their experience. The responses were summarized and collated by A.A. and M.K. and shared with the HCD working group members iteratively to arrive at identified successes and challenges as a group.

## 3 Results

### 3.1 HCD Working Group Sessions

We conducted 31 HCD working group sessions lasting an average of 60–90 min over 12 months (March 2022 and March 2023). These sessions were structured to introduce a new design method, such as journey mapping (Supporting Information S1: Appendix Item 3), followed by practical application. An agenda was created and distributed by the HCD specialist at least 1 week in advance to prepare participants for collaboration. Group members could also suggest items to be added to the agenda before the session over email or at the beginning of each session. The agenda included three key sections: background, methods, and examples. The background section served to align the work of the group with the project's overarching goals and vision. The methods section delineated the current stage of the design process (discover, define, develop or deliver) and introduced the upcoming method, including the purpose and application to extract patients' insights and needs from patient interviews. The examples section provided additional context to better prepare members for the sessions' tasks. Each session began with an introduction and an overview of key information for the session. This was followed by dedicated work time for the material to be addressed that day. This work time typically took place in Mural [26], an online collaborative digital whiteboard, allowing all participants to work simultaneously and collaboratively. The length of work time varied depending on the activity. Afterwards, the group reconvened with cameras and microphones on to discuss comments and recommended changes. Discussions were held on each item/recommendation until a consensus was reached before moving on to the next item. Sessions were recorded and uploaded to a shared folder to accommodate for full participation by all members, including those who could not attend a session. Members could also provide feedback over email if they were unable to attend.

### 3.2 HCD Working Group Outputs

**Figure 2:**
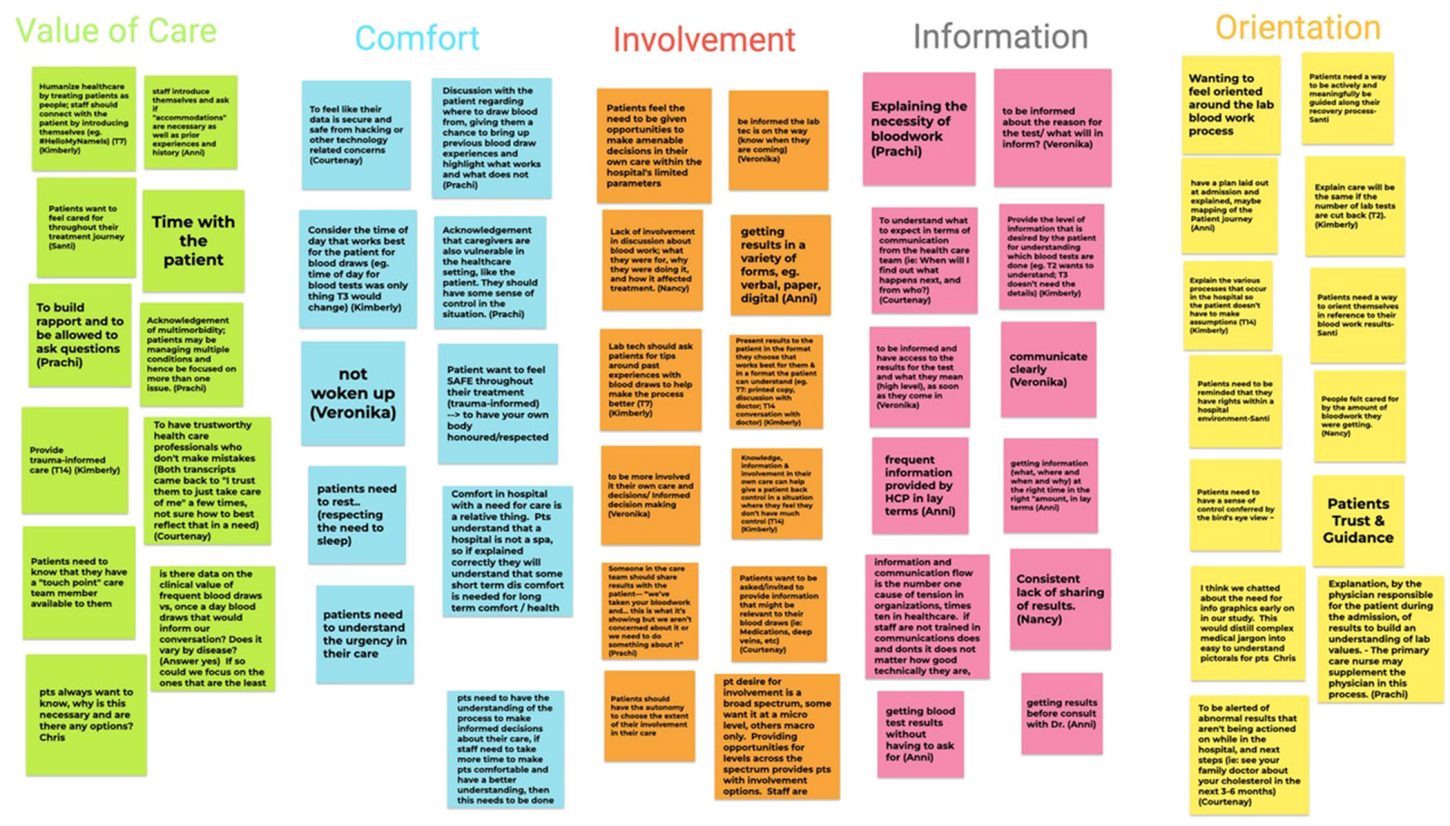
Human centred design working group affinity diagram.

**Figure 3:**
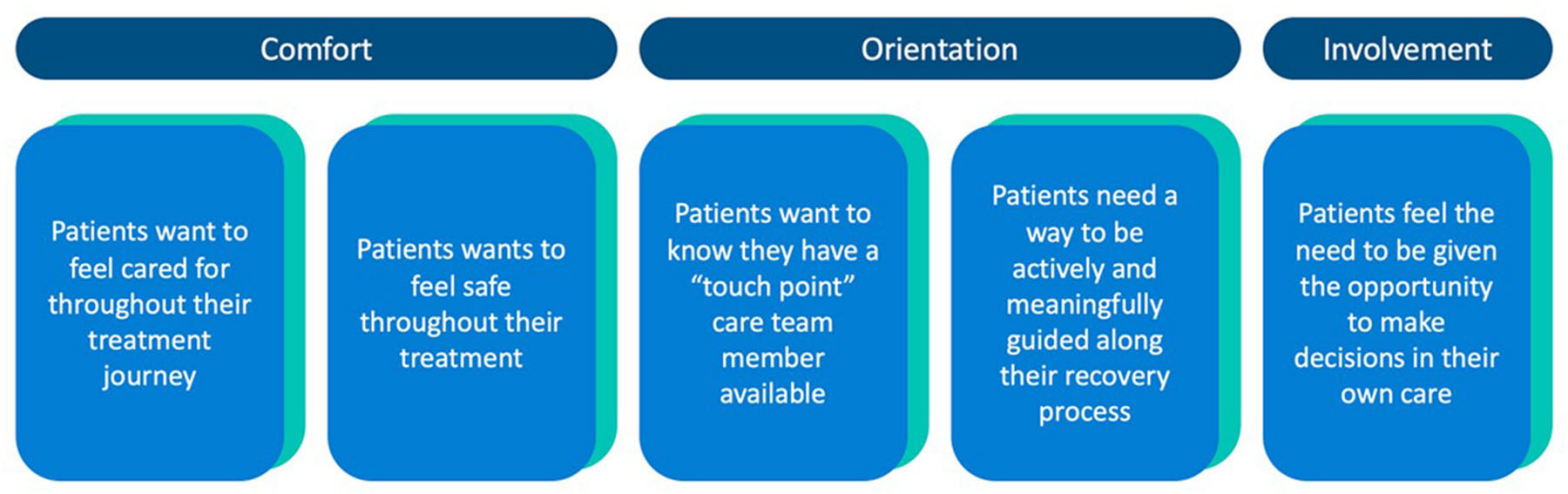
Five key insights identified from the human centred design working group affinity diagram.

The group created a journey map and storyboard (Supporting Information S1: Appendix Items 3 and 4, respectively) to help synthesize the qualitative data from the previously collected patient interviews. The PRPs were trained in rapid analysis to categorize the data to form themes, resulting in an affinity diagram (Figure 2)—a diagram used to organize ideas into emerged categorical relationships [27]. This affinity diagram and the developed themes helped identify major challenges that patients face during the hospital bloodwork process and provided critical insights for the HCD process (Figure 2). Five key insights were derived from the identified themes (Figure 3).

Using these insights, the group co‐developed a design statement: ‘How might we help patients requiring bloodwork make informed care decisions, while actively and meaningfully being supported in a safe and respectful hospital environment?’ This design statement guided the rest of the group's work, leading to the development of three patient engagement tools to address the identified needs of patients. These tools are described below.

**Figure 4:**
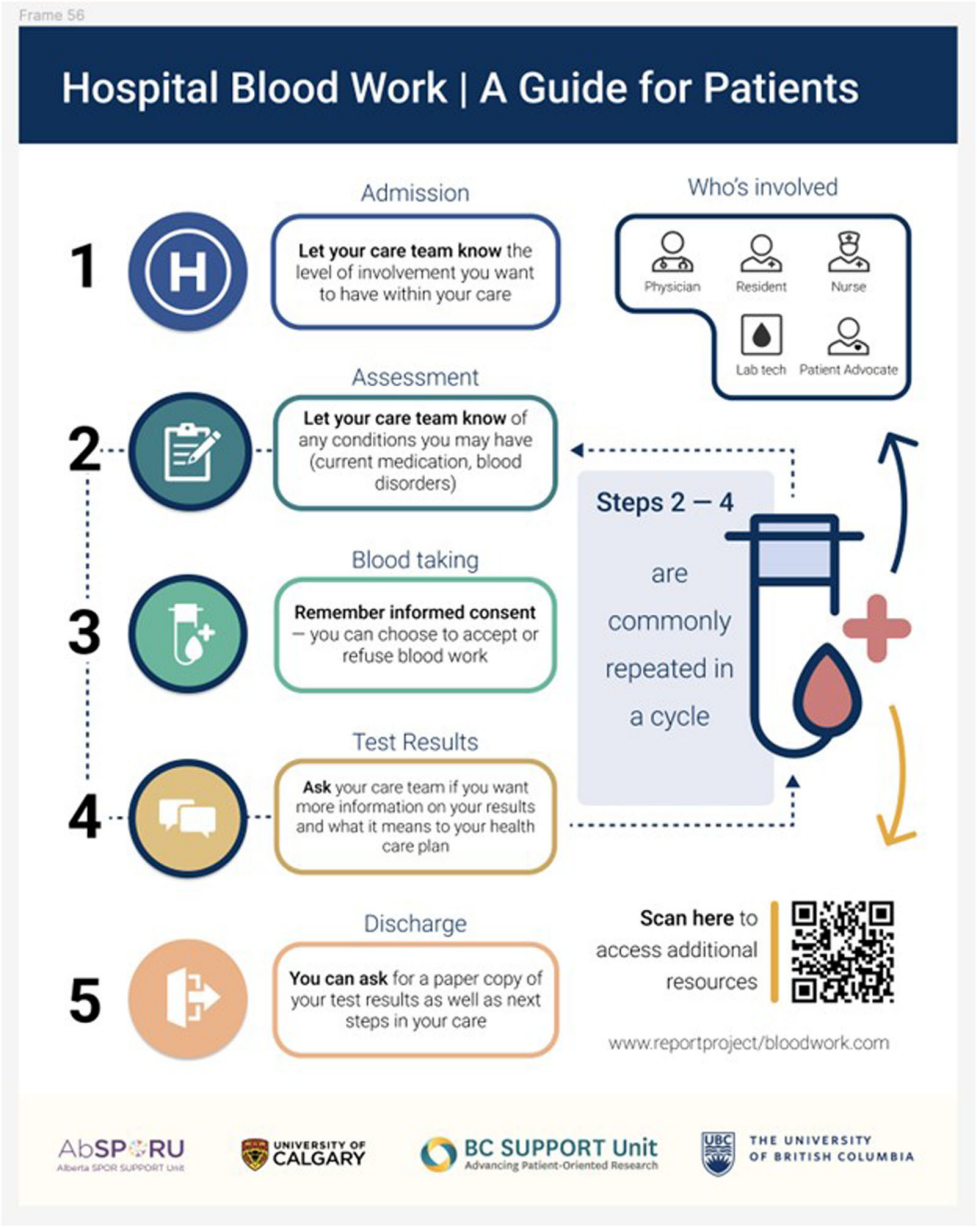
Patient infographic.

*1. Infographic:* The infographic (Figure 4) aims to concisely explain the bloodwork process to patients with little hospital experience. The primary goal of this infographic is to help patients recognize opportunities within the bloodwork process to actively participate in their care, fostering a sense of partnership. The infographic was presented to interview participants for feedback and revised accordingly. The final infographic design was approved by the entire HCD working group and licensed under a Creative Commons Attribution‐NonCommercial‐ShareAlike 4.0 International License [28].

*2. Video:* The video aims to inform viewers about the bloodwork process and is designed to address the questions patient participants raised during the interviews. The video format contains the same steps outlined in the infographic—admission, assessment, blood taking, test results, and discharge—to reinforce what the blood draw process in the hospital looks like. The video introduces real‐life care team members who answer common patient questions about their role in care. PRPs participated as actors in the video in various roles, including that of patients, and spoke from a co‐designed script for the video. The script used anonymized quotes from patients from previous work conducted through the PAC [18].

3. *Website:* The website [29] serves as a central hub for detailed bloodwork information including the process, steps for patient involvement, common hospital blood tests, and additional resources. The website [29] also features the infographic and video, and lists the healthcare providers involved in the bloodwork process and their roles.

### 3.3 Patient Research Partner Engagement Within HCD to Facilitate Co‐Building

The HCD working group operationalized the CIHR patient engagement principles [4] of (1) mutual respect, (2) inclusiveness, (3) and support to facilitate co‐building. *Mutual respect* was demonstrated by bringing together people with diverse and complementary skillsets. PRPs guided the process through the lens of their lived experience, AbSPORU [19] members facilitated PRP engagement, and academic researchers worked with the HCD specialist to support the design process (e.g., facilitate sessions, organize appropriate training for working group members, and synthesize the input throughout the design of the patient engagement tools). *Inclusiveness* was achieved by having a diverse representation of PAC members within the working group. At least six PAC members attended each HCD working group session, and updates were shared at monthly PAC meetings. PRPs offered guidance on wording, design, appropriate imagery, and overall information to include in the patient engagement tools. *Support* was provided through comprehensive HCD training and financial compensation for PRPs. Support was also provided by the AbSPORU [19] Patient Engagement Team members, who played a key role in facilitating meaningful patient engagement within the working group.

### 3.4 Successes and Challenges Encountered

The debrief session was 1 h long and attended by six PRPs and two research team members. Those unable to attend provided feedback via email. The results were organized and iteratively revised through group feedback by A.A. and M.K. (Table 1). All members of the PAC, including PRPs, academic researchers, the HCD specialist, and AbSPORU [19] team members found the overall experience of blending HCD with patient engagement principles to be valuable and stimulating. HCD working group members valued the breadth of experience and knowledge among team members and appreciated the overall culture of respect within the group. Key successes identified by the group included the creation of a diverse and inclusive environment, the availability of learning opportunities on HCD and qualitative research, and the establishment of tight feedback loops. PRPs particularly noted the beneficial role of AbSPORU [19] Patient Engagement Team members in facilitating their active engagement. PRPs reported that they were all able to share their voices and ultimately come to consensus on many decisions.

**Table 1:** Successes, challenges, and opportunities noted by the human‐centred design (HCD) working group.

Successes	Challenges	Opportunities
Including Alberta SPOR Support Unit [19] to enhance patient engagement	Different degrees of experience and comfort with patient engagement knowledge and training amongst research team members	Provide formal patient engagement training to all research team members, including specialists
Blending HCD and patient engagement methodology	Late stages of project became repetitive due to difficulties with consensus‐building	Create project map and communication plan before project start
Welcoming and diverse environment	Difficulty reaching consensus leading to disproportionate time spent among different tools	Implement resolution tracking and create communication plan and transparent consensus‐building approaches
Many learning opportunities provided to patient research partners	Lack of debrief touchpoints throughout project	Implement debrief touchpoints earlier and throughout projects

PRPs noted that the HCD process became repetitive during later stages. This was partly due to difficulties in building consensus, leading to several topics being re‐discussed. In the future, a more transparent communication plan is recommended, which includes a process for reaching consensus and tracking resolved issues. The redundancy in discussions also led to uneven time distribution with more focus on the infographic and less on the video and website. Balancing adequate discussion within the HCD working group with project funding timelines proved difficult, leading some PRPs to feel they did not have sufficient opportunities to fully contribute to the final design of the website and the video. Project mapping and improved management is recommended to reduce redundancy in the HCD process and ensure better facilitation to effectively manage the time allocated for each discussion item. Further, PRPs identified that it would be beneficial for all members of the working group to receive training in patient engagement. Additionally, some PRPs raised concerns about the adaptation of the infographic (and associated changes) by different hospitals.

## 4 Discussion

In this manuscript, we describe how we successfully applied the principles of patient engagement with the steps of HCD to codesign patient engagement tools within our PAC. The experience of our PRPs demonstrates success in operationalizing the principles of patient engagement to facilitate co‐build. Like other studies using HCD and patient engagement principles [1, 8, 30], this study showcases that the two complement each other and produce truly patient‐centred materials. However, while in other studies HCD has been used as a tool in the context of participatory research [5, 6, 31, 32], we are among the first to describe the use of HCD in a patient‐oriented research project with a long‐standing PAC (established in spring 2021). Even in patient‐oriented research projects that use other co‐building tools, the project timeline is usually short and thus, little time is spent on co‐building project outcomes [33]. Our HCD working group spent over 50 h in more than 30 group sessions and workshops, co‐building patient engagement tools over the course of 1 year.

Furthermore, this sustained engagement with the PRPs has fostered a strong bond, facilitating an environment of mutual respect and trust. HCD working group members were comfortable sharing their concerns with the understanding that the team would collectively work through disagreements. As studies have shown, combining patient engagement and HCD requires shared decision‐making [30]. It is also essential to create a safe space for PRPs to share their thoughts [30], which is demonstrated in the honesty of the debrief session held after the working group. This long‐term engagement is essential to the success of HCD, as it has been found that, with the short‐term engagement of communities typically available in the healthcare setting, long‐term change is difficult, if at all possible [34].

Additionally, the RePORT project has demonstrated the ability to successfully operationalize patient engagement principles in previously published work [16, 17, 18]. This study takes another step in demonstrating the successes in operationalizing these principles through using HCD for co‐build, while also identifying opportunities for improvement. All current six PRPs from the PAC contributed to the study and the contents of this manuscript.

We identified many avenues for improvement in the application of patient engagement to HCD in our study. Many learnings were taken from this project, particularly, how important it is to balance patient engagement with consensus building and project timelines. We identified opportunities with respect to transparent sharing of project timelines and consensus building approaches to avoid feelings of tokenism in PRPs. Importantly, the PRPs and research team members agreed that scheduled regular debrief sessions during the project would have been helpful. In the future, regular debrief touchpoints will be scheduled with the PAC during co‐build projects. To retain the originality of co‐built tools while recognizing the requirement of tailoring to local contexts by individual hospitals, the originally developed infographic is displayed on the project website under a Creative Commons Attribution‐NonCommercial‐ShareAlike 4.0 International License [28].

In terms of next steps, the patient engagement tools developed from the HCD working group are currently being evaluated. The PAC co‐developed a survey and interview guide to assess the effectiveness of the patient infographic (with QR code linking to website and video) displayed in hospitals. PAC members will analyse the interviews to identify themes related to the tools' effectiveness and areas for improvement. PRPs have received qualitative analysis training to help with the analysis. The findings will then be presented to the PAC, who will use them to refine the tools to better meet patient needs.

## 5 Conclusion

This manuscript has demonstrated how HCD and patient engagement complement each other and can be effectively operationalized. This study also highlights the outputs that can be generated through collaboration between research teams, PRPs, and HCD specialists. HCD provides structure and opportunities for shared decision‐making and codesign, and is most effective when integrated with clear communication, project mapping to adhere to timelines, and transparent consensus‐building approaches. This study serves as a guide for future researchers on how to operationalize patient engagement within a HCD framework.

## Author Contributions

**Madelyn Knaub:** conceptualization, methodology, writing – original draft, writing – review and editing; formal analysis, visualization, data curation, investigation, validation. **Santiago Leon:** conceptualization, methodology, software, formal analysis, writing – original draft, writing – review and editing, investigation, project administration, visualization, data curation, validation. **Surakshya Pokharel:** project administration, conceptualization, methodology, investigation, formal analysis, writing – review and editing, writing – original draft. **Veronika Kiryanova:** conceptualization, methodology, writing – review and editing, formal analysis, investigation. **Kim Giroux:** writing – review and editing, conceptualization, methodology, investigation, formal analysis. **Prachi Khanna:** writing – review and editing, conceptualization, investigation, methodology, formal analysis. **Anna Rychtera:** writing – review and editing, conceptualization, methodology, investigation, formal analysis. **D'Arcy Duquette:** writing – review and editing, conceptualization, methodology, formal analysis, investigation. **Pamela Mathura:** writing – review and editing, conceptualization, methodology, formal analysis. **Nancy Verdin:** writing – review and editing, formal analysis, methodology, conceptualization, investigation. **Anshula Ambasta:** funding acquisition, conceptualization, methodology, validation, formal analysis, resources, investigation, writing – original draft, writing – review and editing, project administration, supervision.

## Ethics Statement

The research program on laboratory test utilization and the qualitative study to understand patient participant perspectives on laboratory testing were approved by the institutional review board Conjoint Health Research Ethics Board at the University of Calgary (REB 17‐1215 and 20‐0728). Since all current members of the Patient Advisory Council and Human Centred Design Working Group are active and equal members of the research team and consent to the publication of this manuscript, no specific ethics approval was sought for this manuscript.

## Conflicts of Interest

The authors declare no conflicts of interest.

## Supporting information

Supporting information.

## Data Availability

Relevant data are within the manuscript and supporting information files. Original recordings and notes are stored by the research team and available upon reasonable request from the Patient Advisory Council, in accordance with institutional policies and procedures.
