# Supplementary material for: Using Human‐Centred Design to Codesign Patient Engagement Tools With a Patient Advisory Council: Successes and Challenges
Source: Health Expect. 2025 Mar 15;28(2):e70230. doi: 10.1111/hex.70230 (PMC11909472; doi:10.1111/hex.70230)

**Appendix**

**Appendix Legend**

**Appendix Item 1 –** Terms of Reference (TOR)

**Appendix Item 2** **–** Guided Questionnaire for Debrief Session

**Appendix Item 3 –** Patient Journey Map

**Appendix Item 4 –** Storyboard

*Appendix Item 1: Terms of Reference (TOR) for PAC*

Terms of Reference

**Re-P**urposing the **O**rdering of **R**outine laboratory **T**ests (RePORT) **P**atient **A**dvisory **C**ouncil (PAC)

**See Glossary of Terms for definition.*

Timeline: Project funding ends in 2026.

# Background of the RePORT study

The RePORT* study is a patient-oriented research* project which aims to co-design a knowledge user*-informed, healthcare provider* and patient partner* intervention bundle* to help address the overuse of laboratory blood tests in hospitals. This bundle will then be implemented, and its impact on the healthcare system, patients*, and healthcare providers will be evaluated. A patient engagement* strategy will be co-developed by a Patient Advisory Council (PAC) whose terms of reference can be found below.

# PAC Purpose

The aim of the RePORT PAC is to help bring a diverse range of insights, perspectives, and reflections together to collaboratively gain a broader understanding of patient experiences of routine laboratory blood tests in a hospital setting.

The RePORT PAC members will work together to co-design in a range of patient engagement and research activities, for example:

- Informing the research project design
- Reviewing and editing documents
- Engaging in learning exercises
- Participant recruitment
- Co-designing the interview guide
- Assisting with interpreting data
- Co-developing of educational tools
- Creating patient engagement strategies
- Assisting with Knowledge Translation*

In addition to the above activities, the PAC members may choose to be involved in other research-related work, for example:

- Taking part in training opportunities (Thematic analysis, NVIVO, conducting semi-structured interviews etc.)
- Data collection (eg. co-conducting semi-structured interviews, focus groups, etc.)
- Data analysis
- Collaborating on the Human Centered Design approach
- Knowledge translation and dissemination* (e.g report writing, co-authoring, co-presenting on project findings and processes)

# Membership

With the goal of equity, diversity, and inclusion in mind, the PAC will consist of:

- 5-10 patient partners
- Healthcare providers
- Academic research team members*

# Co-chairs

Co-chairs are three members from the RePORT Advisory Council and include two patient partners and one academic research team member. Should one of the co-chairs step down from their duties, the responsibilities shall be redistributed, decided collectively by the remaining members. The roles will evolve according to the needs of the council.

Initially, the patient co-chairs were selected by the Alberta Strategy for Patient Oriented Research SUPPORT Unit (AbSPORU) Patient Engagement (PE) Team* members, and the research team (Phase I). As the project progresses (Phase II and on), patient co-chairs can self-nominate or be nominated by any PAC member. Patient co-chairs are elected by the PAC. Patient co-chair term is up to two years.

Co-chair appointments should be staggered so that there is always an experienced co-chair active in the committee. So, there will be one new co-chair election per year so that the co-chair changes overlap with a current co-chair position.

The co-chairs will be responsible for:

- Drafting and distributing the agenda for meetings
- Facilitating the meetings and discussions
- Ensuring Notes and Action Items and Zoom recordings of the meetings are distributed, and uploaded to the Google Drive (within 3 business days)
- Creating a safe space and allowing everyone to speak and be heard
- Guiding individual council members to work toward a common goal
- Working together as a team to resolve any conflicts and misunderstandings
- Providing support to interested advisory council members to collaborate on research related work, including data collection, analysis, and dissemination of findings

# Meetings and Information Sharing

All meeting materials and other resources will be stored in Google Drive (Link Here). Necessary meeting materials will be emailed, or upon request, mailed as a paper copy. Meetings will be held online over Zoom, with a phone-in option available.

PAC co-chairs will be available to address any questions or concerns. For contact information, see the Membership List (available on the Google Drive)

- The PAC will meet once every 3 months, with additional meetings as required.
- An agenda with relevant documents for the meeting, including previous meeting’s notes, will be developed by the co-chairs collectively, and will be distributed by the Academic Research co-chair at least 3 days prior to the PAC meeting date.
- The Academic Research co-chair will be responsible for scheduling meetings, sending meeting invites and hosting the Zoom session. The other co-chairs will be made co-hosts in case the Academic Research co-chair is sick so the PAC can still meet.
- A recording of each meeting will be made by the Zoom host and housed in the Google Drive.
- Notes and Action Items will be compiled by an academic PAC co-chair and circulated by email to PAC members shortly after each meeting. These will also be housed in the RePORT Google Drive.
- If a PAC member is unable to attend a meeting, please notify a co-chair by email.
- Council members are expected to review the meeting Notes and documents from any missed meetings. Council members are encouraged to watch the video recording of any missed meetings.
- Reviewing items pre-meeting will save time and make for better meetings.
- Majority vote is not required. The PAC will strive to work by consensus in drafting its advice.

#

# Compensation for Time and Reimbursement of Expenses for PAC Patient Partners

- Compensation will be offered, as per AbSPORU Guidelines* at a rate of $25 per hour for:
  - Each meeting attended
  - Compensation for 30 minutes of member's time will be offered for reviewing agendas and attachments prior to meetings.
  - Any work outside of meetings, for example:
    - Any additional preparation and review of documents or other material
    - Any other research tasks or activities outside of meeting hours assigned by the academic research team.
- PAC patient partner members are responsible for tracking, documenting and submitting hours in the member’s Compensation Submission Form (update link expenses incurred when meeting in person). Reimbursement is also given for expenses incurred. Reimbursement expenses may include:
  - Transportation (gas, mileage, parking, bus fare, taxi, airfare, etc.)
  - Food
  - Accommodation
  - Other expenses as approved by the academic research team
- The RePORT Patient Advisory Council (PAC) Research Development Award has been created to support Patient Research Partners (PRP) in attending and presenting at conferences. Further, this award can be used for research-skill development for PRPs. This award is intended to help lessen the financial burden of these activities by covering a portion of expenses.
  - The award amount is as follows for the duration of the project until 2026:
    - Up to $1000 per PRP
    - Up to $1500 per PRP who has served as co-chair
    - An additional $500 can be added if the award applicant is presenting RePORT related work at a conference
  - PRPs need to apply for this award and more information can be found here: Click Here.

# Confidentiality

- All personal information is to be kept confidential.
- Research information is not to be shared outside of the PAC.
- If you are unsure of whether certain information is confidential, consult the principal investigator.

# Stepping Down

Notification must be given to the RePORT PAC co-chairs and the PI (Anshula Ambasta) via e-mail. A patient partner can step down by phone as well but this then needs to be followed-up by written confirmation.

# Review of the ToR

This is a living document. Changes are expected and welcomed as the RePORT project progresses. This document was last edited on July 31, 2024.

**Version History**

Version 1; September 2, 2021 (Link Here)

Version 2; November 15, 2021 (Link Here)

Version 3; January 10, 2022 (Link Here)

Version 4: March 2023 (Link Here)

Version 5: May 2023 (Link Here)

**Glossary of Terms**

*Listed alphabetically.*

**AbSPORU Patient Engagement (PE) Team:** The Alberta Strategy for Patient Oriented Research SUPPORT Unit Patient Engagement Team provides ongoing support to patient, family, and community research partners, academic researchers, and community organizations to collaborate on health research priorities and studies. Enhancing the collaboration and capacity of all health research team partners contributes to meaningfully advancing the science and practice of patient engagement. <https://absporu.ca/patient-engagement/>

**Academic Research Team Member:** Staff members of the RePORT project who conduct research. This includes research assistants, research associates, quality improvement specialists, the principal investigator, and the program manager/research scientist.

**Healthcare practitioner:** A physician or other licensed individual who delivers healthcare services. <https://www.albertadoctors.org/emr_resources/GlossaryofTerms.pdf>

**Intervention Bundle for RePORT study:** The intervention bundle for RePORT includes two arms:

1. Healthcare provider engagement strategy: this includes the use of an educational module, clinical decision support tool, and audit and feedback reports on the utilization of laboratory testing
2. Patient engagement strategy: this will include a patient-created infographic, [website](https://www.hospitalbloodwork.ca/), and video (on the website) to help engage patients with the study and its objectives.

**Knowledge Dissemination:**is the interactive process of communicating knowledge to target audiences so that it may be used to lead to change. The challenge is to improve the accessibility of desired knowledge products by those they are intended to reach. This means ensuring physical availability of the product to as large a proportion of the target audience as possible and making the product comprehensible to those who receive it. <https://link.springer.com/chapter/10.1007/978-981-10-0983-9_97>

**Knowledge Translation:** A dynamic and iterative process that includes synthesis, dissemination, exchange and ethically-sound application of knowledge to improve the health of Canadians, provide more effective health services and products and strengthen the health care system. (*Source: Canadian Institutes of Health Research)*

**Patient:** An overarching term inclusive of individuals with personal experience of a health issue and informal caregivers, including family and friends. (*Source: Canadian Institutes of Health Research)*

**Patient Engagement** : CIHR Strategy for Patient-oriented Research, Patient Engagement Framework
<https://cihr-irsc.gc.ca/e/48413.html>

**Patient-Oriented Research:** A continuum of research that engages patients as partners, focuses on patient-identified priorities and improves patient outcomes. This research, conducted by multidisciplinary teams in partnership with relevant stakeholders, aims to apply the knowledge generated to improve healthcare systems and practices. (*Source: Canadian Institutes of Health Research)*

**Patient Partner:** Individuals representing a variety of experiences, perspectives, and backgrounds who have lived experience of various health conditions and have received routine laboratory testing while in hospital. As active and equal members of the research team, they bring valuable insights and knowledge to the research.

**Patient Recruitment:** Recruitment of patients to the RePORT study includes identifying eligible patients, explaining the study to potential participants, and obtaining informed consent to participate in the program and provide information for research.

**RePORT:** Re-Purposing the Ordering of ‘Routine’ laboratory tests is a study that addresses the problem of laboratory test overuse in hospitals and proposes to develop an intervention bundle comprising a stakeholder-informed healthcare provider engagement strategy, and a patient co-designed engagement strategy. The study will implement this bundle and evaluate its impact on the healthcare system, patients, and healthcare providers.

**Stakeholder**: An individual, group or organization having a “stake” in an issue and its outcome <https://cihr-irsc.gc.ca/e/documents/ce_framework_e.pdf>

**Terms of Reference:** A terms of reference document outlines the ways in which a group of people agree to work together to accomplish common goals *(Source: Health Quality Ontario)*

**PAC Workflow:**

Phase III: Support implementation of intervention bundle across hospitals in AB. Evaluate AB implementation. Quantitative and Qualitative evaluation + dissemination of results.
Examples include: Co-develop interview guide, conduct interviews, and analyze data post implementation
Example includes: Provide advice on the evaluation of co-developed tools i.e. infographic, video, website

(March 2023 to December 2024)

Phase V: Oversee massive implementation of KT tools
(Jan 2025 onwards)

Phase I: Co-develop a patient interview guide, conduct patient interviews and analyze data

(Aug 2021 to June 2022)

Phase II: Use the interview data to create a Patient Infographic, Project Website & Video with a Human Centered Design Specialist

(Feb 2022 to March 2023)

Phase IV: Use implementation data to further edit and update patient engagement/KT tools
(April 2024 to December 2024)

*Appendix Item 2: Guided Questionnaire for Debrief Session*

| Questions |
| --- |
| 1. How was your overall experience? Think big picture here, a quick synopsis before we get into the nitty-gritty. |
| 2. What were some challenges you faced?  Prompt for logistical challenges as well here if not brought up at any point |
| 3. What worked well? What were some things you liked?  Prompt for logistical accomplishments as well if not brought up |
| 4. How was it working with AHS? Did AHS hinder or aide your experience? |
| 5. I noticed that members of SPOR (e.g., Sandra, Ingrid, etc.) were involved in many of the HCD meetings. Did you find SPORs presence enhanced your experience? How did they enhance your experience? |
| 6. Do you think the research team could have done other things to improve the patient engagement aspect? |
| 7. Is there something you would change about the process that led to the outputs? |
| 8. How do you think we could have improved patient engagement on the research side? |
| 9. Are you satisfied with the materials produced? Do you feel the patient voice is present in these materials? |
| 10. What principles of patient engagement do you feel were best met during this process? What principles were the least met? |
| 11. What training did you receive? |
| 12. How do you think this work can be applied to other projects? |
| 13. If you were to do this whole thing all over again with a fresh slate, what are the changes you would make and how would you approach it? |

*Appendix Item 3: Patient Journey Map highlighting the different moments, what works, and challenges within the bloodwork process identified by the PRPs from patient interviews*


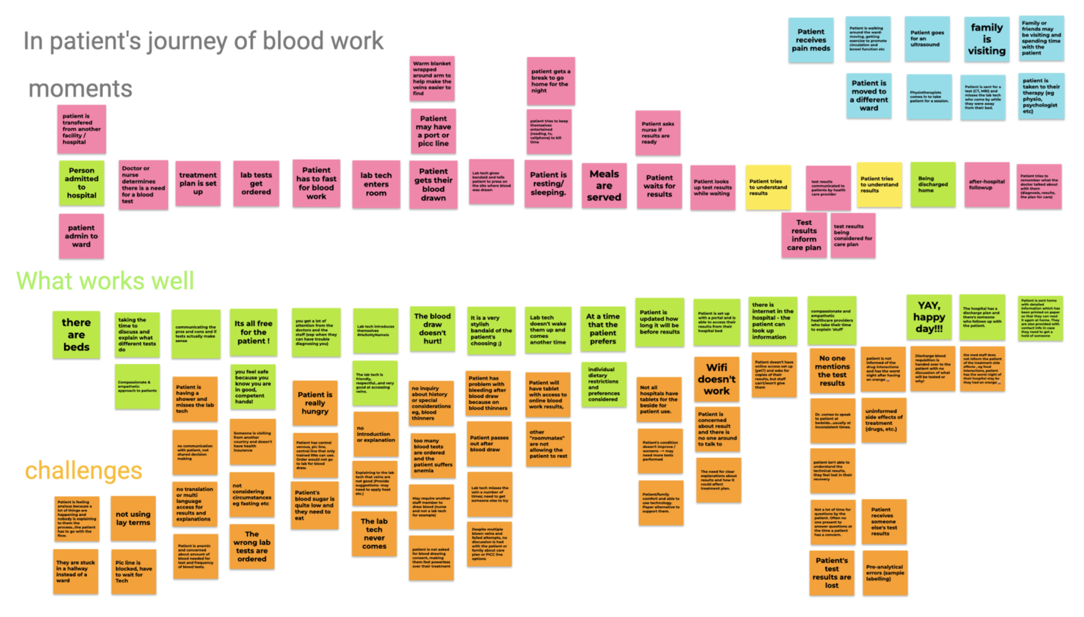


*Appendix Item 4: Storyboard highlighting challenges and needs identified from Rapid Analysis*


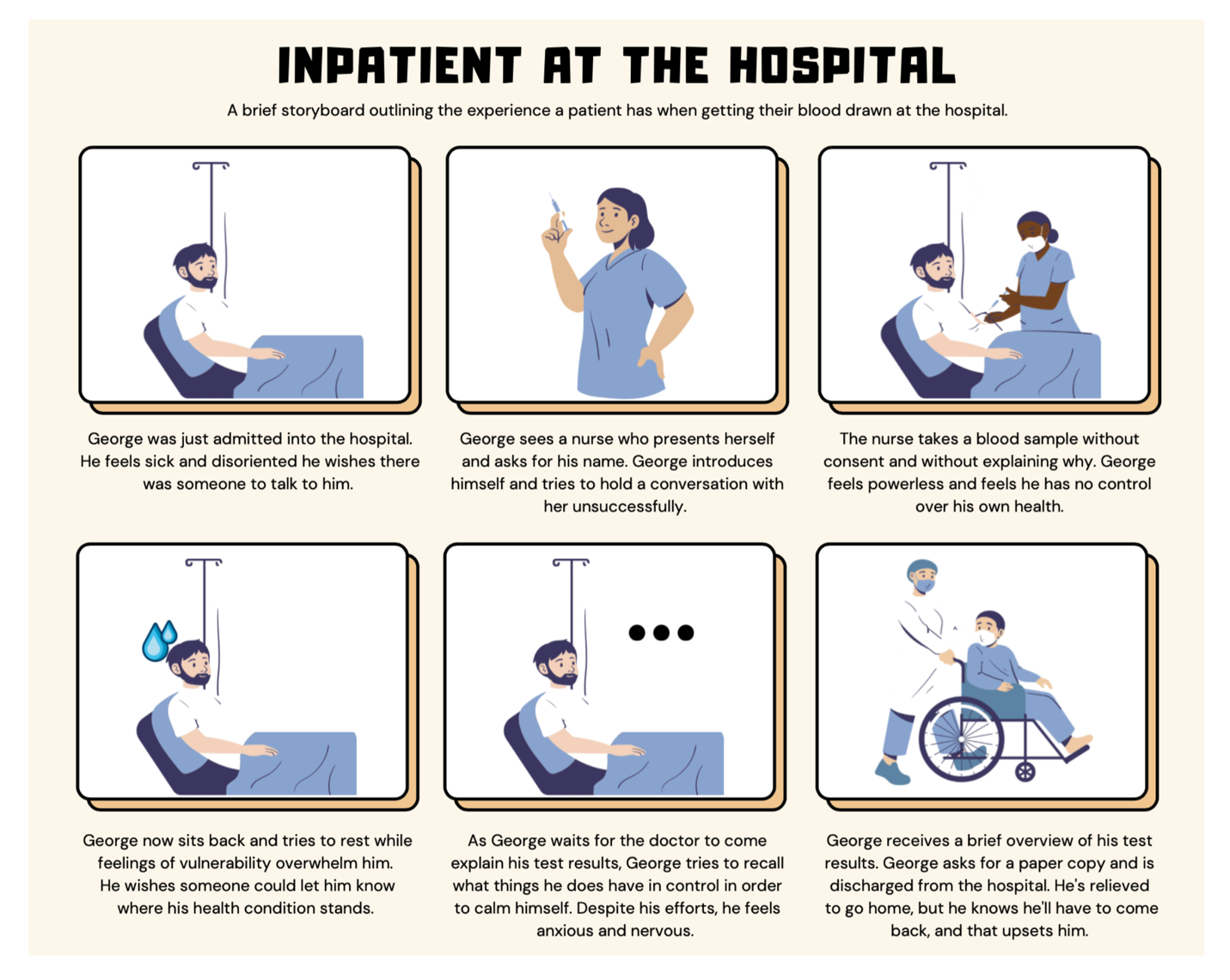

Supplement: Supplementary file 1 — Supporting information. [file HEX-28-e70230-s001.docx]
